# Molecular Basis for Evolving Modularity in the Yeast Protein Interaction Network

**DOI:** 10.1371/journal.pcbi.0030226

**Published:** 2007-11-09

**Authors:** Ariel Fernández

**Affiliations:** 1 Department of Bioengineering, Rice University, Houston, Texas, United States of America; 2 Department of Computer Science, The University of Chicago, Chicago, Illinois, United States of America; University of California San Diego, United States of America

## Abstract

Scale-free networks are generically defined by a power-law distribution of node connectivities. Vastly different graph topologies fit this law, ranging from the assortative, with frequent similar-degree node connections, to a modular structure. Using a metric to determine the extent of modularity, we examined the yeast protein network and found it to be significantly self-dissimilar. By orthologous node categorization, we established the evolutionary trend in the network, from an “emerging” assortative network to a present-day modular topology. The evolving topology fits a generic connectivity distribution but with a progressive enrichment in intramodule hubs that avoid each other. Primeval tolerance to random node failure is shown to evolve toward resilience to hub failure, thus removing the fragility often ascribed to scale-free networks. This trend is algorithmically reproduced by adopting a connectivity accretion law that disfavors like-degree connections for large-degree nodes. The selective advantage of this trend relates to the need to prevent a failed hub from inducing failure in an adjacent hub. The molecular basis for the evolutionary trend is likely rooted in the high-entropy penalty entailed in the association of two intramodular hubs.

## Author Summary

The protein interaction network or interactome emerged as a powerful descriptor in the large-scale phenotypic studies of the post-genomic era. A major concern in such analysis is the integration of interactomic information with other phenotypic descriptors such as expression profile, co-localization, developmental phase, and large-scale protein–structure data. The latter aspect of the integration is the focus of this contribution. We investigate the molecular basis of network robustness to node failure in the most thoroughly characterized interactome, the yeast network. Node failure is by no means a random occurrence across the network as often claimed, but likely to arise in the node-proteins which are structurally the most vulnerable, that is, the ones most prone to misfolding and to form aberrant associations, including aggregates. Thus, network robustness mandates that such nodes not be directly connected, as failure in one hub is likely to induce failure in an adjacent hub. This observation led us to investigate the molecular basis for the avoidance of connections between highly central proteins and to delineate the graph topology resulting thereof. We show how this topology arose in present-day networks and how it differs from the more generic emerging topology of the ancestral network.

## Introduction

Scale-free networks have been proposed as universal models to describe diverse complex systems such as the Internet, social interactions, and metabolic and proteomic networks [1,2]. The scale-free “topology” is defined by a power-law distribution: A(n) ∝ n^−γ^, where A(n) is the abundance of n-degree nodes and *γ* is a positive exponent. It has been recently noted that such a generic definition does not determine a unique graph topology [3,4]. Rather, topologies ranging from the assortative [3,5], with frequent like-degree node connections, to the highly dis-assortative [5], with like-degree nodes avoiding each other, may fit the same connectivity scaling law [3]. In a purely operational sense, a highly self-dissimilar network is hereby regarded as modular in the sense that high-degree nodes tend to avoid each other [6], and, thus, highly interconnected regions are loosely connected to each other. The definition hinges on the assumption that highly interconnected regions are organized around hubs (the nodes with high degree of connectivity) which would be then characterized as intramodular [3,4].

To determine the graph topology of the yeast protein network [6–10] beyond the power-law distribution and its evolution from a primeval network, we make use of a metric indicative of the degree of graph modularity [3]. The metric is informative of network structure because it increases with the frequency of like-degree connections, and decreases as the graph topology approaches a modular organization in the sense defined above. It should be noted that there is no inherent contradiction in having a scale-free network endowed with a modular topology that reflects a self-dissimilar or dis-assortive structure, since the characterization of scale-free network is solely based on degree distribution [3,6,9].

We found that the present-day network is actually a self-dissimilar graph, most often linking nodes of dissimilar degrees, thus revealing a marked avoidance of intramodular hub connections in accordance with previous observations [6]. By contrast, ancestors of the network obtained through orthologous categorization of the yeast open reading frames (ORFs) [8] are progressively more assortative as we regress toward the network of ancient proteins. The assortative topology brings the ancient network closer to a physical system, where assortativity becomes a generic attribute of the statistical mechanics of phase transitions, and thus an emerging property more readily attainable than modularity [11].

The robustness of the present-day network is found to differ from typical scale-free attributes, since it minimizes its vulnerability to hub failure and not to random node failure [2], with the former being more likely in protein interaction networks, as shown below. The evolution toward self-dissimilarity is shown to be reproducible through propagation laws of connectivity accretion that promote progressive increase in modularity. Finally, the molecular basis for the observed trend toward a scarcity of like-degree node connections is delineated.

## Results

### A Graph Metric to Monitor the Evolution of Modularity

The metric S(G) (0 ≤ S(G) ≤ 1), for a graph G with scale-free degree distribution is defined by [3]:

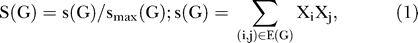
where E(G) is the set of graph edges, (i, j) is a generic edge linking nodes i and j, X_i_, X_j_ are the respective node degrees (connectivities), and s_max_(G) is the maximum over all s(H)-values, where H is a graph with the same connectivity distribution as G obtained by connectivity rewiring. This distribution-preserving rewiring is constructed following [3,6].

For a given scaling degree distribution, the metric is informative of the graph structure, reaching its maximum value (S(G) = 1) in the case where edges are most frequently connecting similar-degree nodes and decreases as the frequency of dissimilar-degree connections increases [3,6]. Thus, a low S(G)-value is indicative of graph modularity in the sense defined above, because the expected frequency of hub–hub connections is low and because connections involving hubs are always dominant contributors to the sum defining S(G) (Equation 1).

Using this metric, we determined the modularity along the natural evolution of the yeast protein interaction network. Node ancestry classes are defined through orthologous representativity in other genomes informative of the yeast evolution (Methods). Ancestry classes are labeled using binary vectors [8] and defined based on the existence of orthologs in other fungi (00011) (36% of yeast proteome), in all other eukaryotes diverging earlier than fungi (00111) (19%), in eubacteria (01111) (9.5%), in archaea but not in eubacteria (10111) (8%), in all ancestral groups (11111) (3.5%), and exclusively in yeast (00001) (24%). Thus, a binary vector denotes an ancestry class of proteins. The ancestry is given by the extent of ortholog representativity. Thus, the binary vector indicates from the right entry (yeast) to the left (progressively more distant life domains) the ortholog representativity of the proteins, with nth entry = 1 if an ortholog of the protein exists in life domain n, and = 0 otherwise. Thus, the network evolution from the ancient-protein (11111) network is retraced by trimming the present-day network through progressive removal of ancestry classes, starting with the most recent (00001). Although the network still contains false-positive and false-negative data in spite of state-of-the-art curation (Methods), the impact of these factors is likely randomly distributed across classes [8] and thus will not significantly affect our conclusions.

The trimming of the present-day network following the schedule imposed by ancestry is based on the assumption that a gene arising at a certain point in evolutionary time in an ancestral organism will be detectable in all species diverging thereafter. The ancestry of a yeast protein is thus defined by the number of orthologous ORFs [8,12]. Thus, no effort is placed in our study in reconstructing the ancestral sequence, a daunting task at the proteomic scale, but rather in assessing its ancestry by genomic comparison. Gene loss or interaction loss due to deleterious evolutionary pressure is possible after speciation, although very difficult to assess and typically neglected in related evolutionary models [8,12].

**Figure 1:**
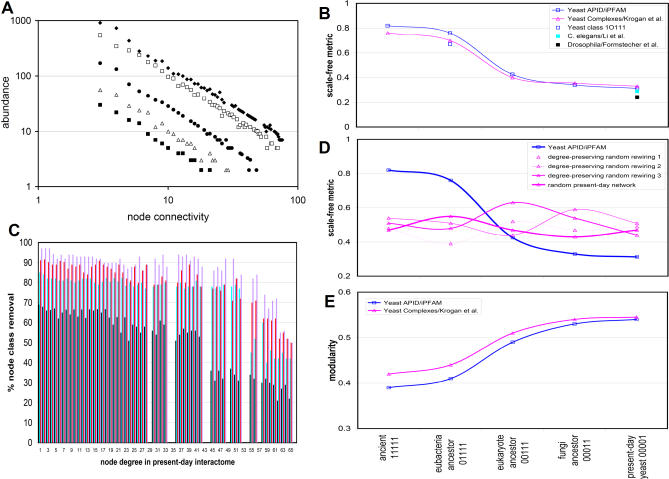
Yeast Network Evolution (A) Scale-free generic law fitting the present-day and ancestral yeast protein interaction network. Abundance of nodes as a function of connectivity (node degree) in log–log scale for the present-day yeast network 00001 (filled diamonds); the fungal ancestral network 00011 (open squares); the eukaryotic ancestral network 00111 (filled circles); the eubacterial ancestor 01111 (open triangles); and the ancient network 11111 (filled squares). (B) Scale-free metric S(G) (blue line plot) indicating the actual graph modularity of the present-day and ancestral networks. Present-day data was cross-validated with the APID database and filtered through iPfam representativity (Methods). The topology best approximated by a scale-free assortative graph (S(G) = 0.82) is that of the primeval network, restricted to the (11111) ancestry class. This ancestral network possesses the emerging properties of assortativity and hub-like core since the large S-value implies that hubs are highly interconnected. This network closely recapitulates typical scale-free attributes. The other ancestral networks were obtained by progressive trimming of the present-day network through exclusion of ancestry classes. Two networks are possible by incorporation of class (01111), with orthologs in eubacteria but not in archaea, or class (10111). Incorporation of the latter class introduces a more pronounced decrease in S-value, implying a scale-richer network. A monotonic trend toward an increase in scale-richness (progressively lower S-values) is apparent. Thus, the network becomes progressively more resilient to hub failures as more recent ancestry classes are incorporated. Notice the dramatic enhancement of self-dissimilarity concurrent with eukaryotic divergence. The calculations using orthologous trimming were repeated using the database of yeast protein complexes of Krogan et al. [14] (magenta plot). The S(G) computations on present-day interactomes for C. elegans [15] (light turquoise blue) and *drosophila* [16] (black) were added for comparison. (C) Percentage removal of nodes with each orthologous trimming iteration. Nodes are grouped in present-day connectivity classes. Node removal is indicated for removal of class (00001) (black), (00011) (light blue), (00111) (red), and (01111) (lilac—light purple). The nodes retained after the final iteration amount to 3.5% of the present-day proteome size. (D) Evolutionary trend toward higher modularity in yeast network (blue line) contrasted with topological evolution of randomly rewired versions of the present-day network (magenta plots). Random rewiring is of two types: degree-preserving and fully random (thick line). (E) Topological evolution of the yeast network characterized by Newman's modularity parameter Q.

The present-day and ancestral networks all fit the scale-free connectivity scaling (Figure 1A). However, their graph topologies are radically different. The ancient protein network possesses a high probability of connection between similar-degree nodes, as indicated by the large S(G)-value, and thus, it is significantly scale-free and assortative. This topology evolved into the scale-rich self-dissimilar graph (S(G) = 0.32) found at the present time (Figure 1B). In contrast with its ancestors, the present-time network tends to connect higher-degree nodes to lower-degree ones, as revealed by the low S(G)-value. Thus, while the ancestral network is actually endowed with the “emergent” properties commonly ascribed to scale-freeness [1,2], such as robustness to random failure, assortativity, and hub-like core, the present-day network is far less generic, more modular [9], and more robust to hub failures. This is evidenced by the dearth of inter-hub edges subsumed in its lower S(G)-value. The selective advantage of this trend relates to the need to prevent a failed hub from inducing failure in an adjacent hub, as shown below.

There are 319 nodes with a present-day degree X > 8 incorporated along the evolution of the network that starts at the ancient network (cf. [8]). All such nodes may be characterized as intramodular hubs [13] that avoid each other and make up for the increased level of scale-freeness in the network topology (Figure 1B). The molecular basis for this like-degree avoidance is described below.

We tested the sensitivity of the results to persistent noise in interactomic data (see Methods for curation details). Thus, in Figure 1B, we contrasted the previously reported behavior of the scale-free metric against the results from progressive trimming of a comprehensive interactome of protein complexes in which ephemeral interactions and high-throughput artifacts have been filtered out [14]. The S-values differ by less than 9% along the entire evolutionary span. Furthermore, the trend toward higher modularity (lower S-value) appears to be commensurate with organismal complexity (Figure 1B), as we incorporate the S(G)-values calculated for the interactomes of Caernohabditis elegans (worm) [15] and *drosophila* (fruit fly) [16].

The dynamics of node removal associated to the evolutionary regression is indicated in Figure 1C, where the percentage of node removal associated with each of the four successive trimming iterations is computed for each node connectivity class in the present-day network. The node removal becomes more severe for the nodes of low connectivity and less pronounced as we approach a higher degree of centrality, in accord with the likely higher level of ancestry of high-degree nodes [17].

The trend toward increasing modularity associated with evolutionary change was further validated by disproving the null hypothesis that this trend holds irrespective of network topology. Thus, in several computer experiments (cf. [3,6]) we randomly rewired the present-day network while preserving the present-day node-degree distribution indicated in Figure 1A. We then successively trimmed the rewired networks following the orthologous classification scheme and computed S(G)-values corresponding to the successive trimmings. The results are shown in Figure 1D. We clearly see that the monotonic and dramatic increase in modularity observed for the real yeast network along the ancient → present-day evolution is not a generic network property, but very much depends on the specifics of the network topology that subsume the biological information. Alternatively, we also randomly rewired the present-day network this time without preserving the degree distribution and randomly and successively trimmed it, removing an equal number of nodes as in the orthologous classification procedure. Again, no trend toward decreasing modularity could be associated with the trimming or, conversely, no clear trend toward increasing modularity is found upon network growth.

### Evolutionary Trend Described by a Measure of Modularity

An alternative indicator of modularity put forth by Newman [18] has been also utilized to better describe the evolutionary trend. Newman's approach not only provides a measure of topological dissimilarity but also identifies or separates the dominant or tightest module, and ultimately—through iteration of the separation procedure—provides a modular partition of the network. The initial modular partition of the network is dictated by the spectrum of a symmetric graph-related matrix. Thus, the dominant moduleM℘ is associated with the largest positive eigenvalue, λ_1_, of the symmetric matrix **B** defined as:

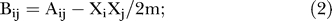
where **A** is the adjacency matrix describing the edge set E(G) (A_ij_ = 1 if nodes i and j are connected, A_ij_ = 0 otherwise) and m = ½Σ_j_X_j_ is total number of edges in the network. The dominant moduleM℘ is univocally defined by the characteristic function χ_M℘_(j) = ½(s_j_(**u**_1_)+1), where **u**_1_ is the eigenvector of **B** associated with λ_1_ and s_j_(**u**_1_) = 1 if the j-th coordinate of **u**_1_ is positive and = −1 otherwise. In set-theory notation: χ_M℘_^−1^({1}) =M℘. This constructive procedure reveals the most densely connected group of nodes with only sparser connections to the rest of the graph and may be further iterated on G\M℘, etc., until a full modular partition of G is achieved. A similar definition of the module is provided in [10].

A modularity parameter Q is then defined as an indicator of the number of nodes falling within modules minus the expected number for a random rewiring of the network, normalized to the total number of nodes in the network. Thus, Q is given by:

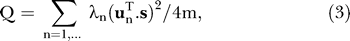
where the dummy index n ranges over all eigenvalues, **u**_n_^T^ is the transposed eigenvector of **B** associated with eigenvalue λ_n_, and **s** = (s_j_(**u**_1_)).

The trend toward increasing modularity associated with evolutionary change in the yeast network evolution is then verified adopting the Q-measure, as shown in Figure 1E: in the ancient network, 39% of the nodes were contained in a module and this number increases to 54% in the present-day network. The dominant module in the ancient network comprises all its 19 ribosomal proteins (see also Protocol S1). This network prevails until class 00111 is incorporated, at which time the signaling module dominates and prevails as dominant in the present-day topology.

### Algorithmic Approximation to Network Evolution

The topological differentiation resulting from connectivity accretion concurrent with progressive incorporation of node classes in the order (11111) → (01111) → (00111) → (00011) → (00001) may be algorithmically reproduced. Thus, the primeval network of ancient nodes–proteins may be abstractly developed, i.e., without reference to concrete molecular features of the node, in a manner entirely consistent with the S(G) behavior shown in Figure 1B.

The algorithmic behavior of network evolution is determined by the probability P(X_n_) = G(n)p(X_n_) that node n with degree X_n_ would acquire a new connection. The p-factor is associated with the rate of connectivity development, while G penalizes like-degree connections that would increase assortativity. The p-factor relates to a preferential attachment law [1,17] in the sense that the probability that a node develops a new connection depends on the number of its pre-existing connections, satisfying:

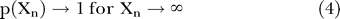



Two accretion laws have been investigated. While heuristic in nature, their accurate reproduction of the evolving network topology makes them worthy of examination:

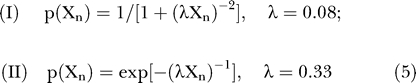



**Figure 2:**
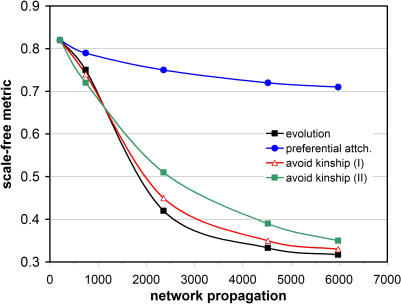
Algorithmic Model of Yeast Network Evolution Natural evolution (black) of the protein network compared with algorithmic network developments (red, blue, green) starting with the network of ancient proteins (node class (11111)). Three algorithmic network developments were computed, following preferential attachment (blue), and laws of connectivity accretion (I in red, II in green) subject to penalization for connection accretion within node kinships.

Both laws have optimized parameters (Figure 2) and satisfy the limit Equation 4.

To prevent similar-degree node connections, nodes are “tagged for kinship” at every stage of network propagation taking into account the order assigned at that stage. This order is obtained by preserving the order arbitrarily assigned in the primeval network while incorporating new nodes in consecutive order.

To define the accretion rules algorithmically, let n_1_ < n_2_ < … be an ordered set of nodes at a specific time in the network development; G_n_ denote the n-centered subgraph, that is, a subgraph containing node n, all nodes connected to n, and the connecting edges; C(n) = {nodes connected to n}; and {G_n_} is a minimal covering of G satisfying G = **∪**_n_G_n_. Then, we may define ξ_n_ = Minimum_n′∈C(n)_ |X_n_ − X_n′_|. Node n is “tagged for kinship” with probability exp(−ξ_n_) provided no node n′ ∈ C(n) with n′ < n has been tagged for kinship. A node n tagged for kinship at a particular stage of network development is assigned the kinship penalty factor

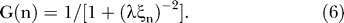



In case of close kinship (ξ_n_ = 0), we get G(n) = 0. The creation of an internal connection linking node n with another node already tagged to develop a connection is governed by probability

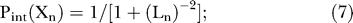
where L_n_ = Maximum_n′∈A(G)_ |X_n_ − X_n′_|, and A(G) = nodes tagged to develop a connection at the particular stage of network development. If node n is tagged to develop a connection, and an internal connection develops, then the new edge connects n to existing node n*, with the latter satisfying: n*∈A(G); L_n_= |X_n_−X_n*_|.

The algorithmic network development that best fits natural evolution (Figure 2) is given by accretion law (I) modulated by precluding kinship connections according to Equations 4 and 5. While law (II) also produces a good fit, it does not portray the sigmoidal behavior of S(G) followed by natural evolution. Network development with an accretion law reflecting preferential attachment (G(n) ≡ 1, law (I)) does not significantly increase its self-dissimilarity relative to the differentiating algorithms that enhance modularity.

### Molecular Basis for Topological Self-Dissimilarity

What sort of selective advantage is associated with evolving toward higher self-dissimilarity or dis-assortativity? We shall show that this trend increases resilience to node failure which is not random, contrary to general assumption [2]. We first note that node failure may result from a loss of the functionally competent structure in favor of a misfolded state. The latter tends to aggregate into a generic aberrant state dominated by the backbone generic information, rather than by the side-chain information that encodes for the native state [19,20]. We cannot assert that misfolding is the sole reason for node failure but it certainly appears to be the dominant one in the light of the results presented below.

**Figure 3:**
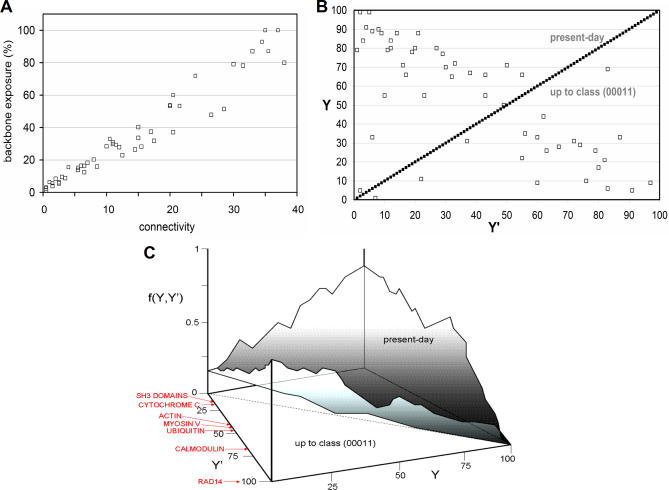
Wrapping, Connectivity, and Ancestry of Yeast Proteins (A) Extent of backbone exposure in yeast proteins from present-day class \(11111) correlates with node connectivity. Backbone exposure is given as percentage of full contour length of the protein (Methods). (B) Distribution probability of connections between yeast proteins in present-day class \(11111) either reported in PDB or natively ordered (Methods) with backbone exposures Y and Y′. The present-day class \(11111) is represented in the region Y > Y′ and the older network up to class (00011), in the region Y′ > Y. (C) Connections between yeast PDB proteins in present-day class \(11111) with backbone exposure levels Y and Y′. Each connection is represented as a point in the Y-Y′ plane, revealing that backbone exposures are significantly anticorrelated across protein–protein interactions.

Soluble proteins with high levels of backbone exposure are prone to aberrant aggregation [20], and thus likely to “fail” since they would be removed from their normal interactive context by relinquishing their native fold. Since, as shown in Figure 3A, intramodular hubs possess a higher extent of backbone exposure in their native soluble structure (the extreme case of this exposure is represented by native disorder) [16,20,21], we may conclude that failure propensity likely correlates with centrality, at least in intramodular organization.

This finding prompts us to ask the question: Why would the avoidance of hub–hub connections bring about resilience to hub failure? Since hubs are characterized by their extent of backbone exposure, they are highly reliant on binding partnerships to preserve their structural integrity [16]. Thus, by distorting its protein–protein interface, a misfolded binding partner is likely to promote the hub failure. Hence, *to prevent a failed hub from inducing failure in another hub, it becomes necessary to minimize the probability that the binding partner of a hub is also a hub*. This is precisely the trend reported in Figure 1B.

Thus, we showed that, unlike robustness to random failure, present-day resilience to hub failure is a non-emergent evolutionary trend achieved by enhancing the dis-assortativity of the graph under the generic scale-free degree distribution (Figure 1A and 1B). Hence, the widespread notion that scale-free networks are vulnerable in this sense does not hold in this particular case.

The lower level of connectivity among nodes of similar degree in the present-day network [6] has a molecular basis that may be delineated and prompts us to invoke conformational entropy penalties. As indicated previously, there are 319 present-day hubs incorporated along the evolution of the network. Of such nodes, 37 are represented in PDB complexes (Protocol S1) and shown to contain an extent of backbone exposure in over 50% of the molecule (Methods). Typically, high intramodular centrality implies that protein associations entail considerable induced fit, since the extent of backbone exposure of such hub proteins is significant and thus so is their conformational plasticity [16,21]. To quantify this trend, we established a correlation between present-day connectivity and extent of backbone exposure on PDB-reported proteins incorporated to the ancient network (Figure 3A, Pearson correlation coefficient r = 0.78). This class of nodes is the complement in yeast proteome of class (11111), and thus it is denoted “\(11111)”. We now examine the molecular characteristics of the associations involving proteins in class \(11111), that is, in the complement of the set of oldest proteins, or in the set of proteins incorporated to the ancestral network. This analysis is needed to rationalize the topological difference between the ancient and present-day network.

Induced fit entails a considerable entropic cost associated with the structural adaptation, decreasing the stability of the protein complexes [19]. Thus, induced fits form in the ephemeral complexes typically found in signal-transduction events. On the other hand, a prohibitively high entropic cost would make it unlikely that protein associations would occur if *both* partners must undergo induced fit. This is reflected in the probability distribution f(Y, Y′) of binding partnerships between pairs of proteins in class \(11111) with backbone exposures Y and Y′ (f(Y, Y′)dY′ = probability of connections between proteins with backbone exposure Y and proteins in the range [Y′, Y′ + dY′]). Proteins with high backbone exposure typically associate with those with low backbone exposure, in an anticorrelated manner (Figure 3B and 3C). Thus, direct comparison of Figures 2 and 3B–3C reveals that *high degree nodes in class \(11111) are unlikely to connect with nodes of comparable degree because of the high entropic cost associated with two concurrent induced fits*. *This anticorrelation (Pearson coefficient r = −0.69) provides a molecular basis for the modularity and self-dissimiliarity of the present-day network*.

**Figure 4:**
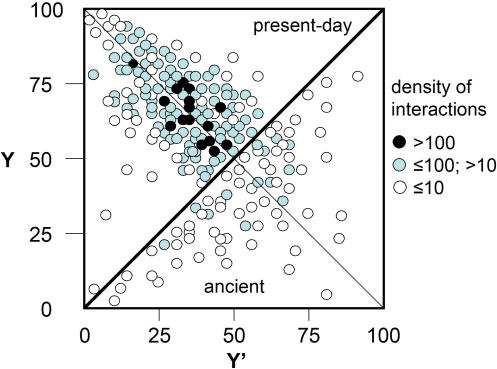
Wrapping Anticorrelation of Pairwise Connected Yeast Proteins Connections from the full yeast interactome plotted in the Y-Y′ plane of sequence-based predicted backbone exposures (Methods) for interacting protein partners in the present-day class \(11111) (Y > Y′) and ancient network (class (11111), Y < Y′).

To extend the validity of the anticorrelation to the full class \(11111), we also adopted a sequence-based predictor of backbone exposure, taking advantage of a tight correlation [16] between extent of backbone exposure and native disorder content, and of the fact that the latter may be predicted directly from sequence [21] (Methods). As backbone exposure in hubs from class \(11111) increases to accommodate interaction partnerships in the evolving network (Figure 3A), their likelihood of mutual interaction decreases. This trend is reflected in the present-day Y-Y′ anticorrelation (r = −0.72) for class \(11111), which evolved from a Y-Y′ correlation (r = +0.66) in the ancient network (Figure 4). This qualitative change reflects the increasing entropy cost of the reciprocal induced fits required to establish hub–hub associations in the proteins incorporated to the ancient class. Thus, the qualitative evolutionary change described at the molecular level (Figure 4) fits the network's seemingly algorithmic progression toward modularity.

## Discussion

Using a metric to quantify the extent of modularity, we examined the evolution of the yeast protein network and found significant topological differences along evolutionary time that reflect a considerable increase in modularity concurrent with evolutionary change. Thus, aided by orthologous node categorization to trace network evolution [8], we established a trend from an “emerging” assortative network [5] to the present-day modular topology [3]. This evolution implies a progressive enrichment in intramodular hubs that avoid each other (cf. [6]), thus increasing resilience to hub failure. This trend is algorithmically reproducible through a network-growth law that disfavors like-degree connections.

The molecular basis for the evolutionary trend toward higher modularity is rooted in the high-entropy cost of the reciprocal induced fits arising from the association of any two intramodular hubs, an event likely to entail structural adaptation in both proteins. Thus, the avoidance of like-degree of nodes of high connectivity is directly related to the extent of backbone exposure and conformational plasticity of hubs, making it entropically costly for them to adapt to binding partners.

This molecular justification of modularity may be complemented by an evolutionary observation. As shown in [8], proteins tend to interact with partners with the same level of ancestry more frequently than with those outside their ancestry class. Thus, the probability that an ancient hub from class (11111) interacts with another hub from the same class is higher than the probability that it would interact with a more recent hub. This effect may in part account for the higher assortativity of the primeval network and for the evolutionary trend toward higher modularity reported in this work. However, a countereffect is also apparent since, by the same token, the probability that a hub from class (11111) interacts with a low-degree node in the same class is also higher than the probability that it interacts with a low-degree node from a more recent class. The relative contribution of each effect is actually subsumed in the computation of evolving modularity reported in this work.

In an alternative molecular approach [22], it was proposed that the number of interactions of a protein is proportional to the number of exposed hydrophobic residues on its surface. This finding would imply that hubs would need to be so hydrophobic that they would hardly qualify as soluble proteins or they would need to be enormous to accommodate all of their binding partners. Furthermore, if this were the case, hub–hub connections would be highly favored through hydrophobic associations, while in known networks this is clearly not the case [6]. Rather, the structural or molecular characteristic of intramodular hubs [17,21] and the attribute that enables them to avoid each other in the network is their likelihood of conformational plasticity and—in the extreme case—native disorder, as demonstrated in this work.

Lacking expression, localization, and developmental coordinates, the protein interaction network provides an incomplete large-scale description of protein–protein associations. Such a study would likely require integration of the interactome and the transcriptome. Thus, the avoidance of like-degree hub connections shown in this work may often materialize in a lack of spatial or temporal correlation between the nodes, a subject of forthcoming work.

## Methods

### Network trimming based on node ancestry classes.

Ancestors of the present-day yeast network were obtained by progressive trimming realized through exclusion of node ancestry classes [8]. Node ancestry classes were determined based on across-species ortholog grouping of yeast proteins. Thus, the primeval network is restricted to nodes with orthologs in all domains of life, while the present-day network incorporates all yeast proteins regardless of their level of ancestry. In a preliminary network curation, connections in the present-day network were only included if independently identified in two sources: Comprehensive Yeast Genome Database from the Munich Information Center of Protein Sequences (http://mips.gsf.de/proj/yeast/CYGD/db/index.html) [23], and reliable subsets of high-throughput screening data [24]. In a second level of curation, the data collected was cross-validated using the APID database that integrates five different repositories for protein interactions including more up-to-date two-hybrid high-throughput data [25]. Finally, the interactomic data was filtered through iPfam representativity (homologous PDB interactivity) [26]. We used iPfam as a database of structurally reported interactions and mapped all interacting Pfam domains onto yeast ORFs using the HMM (hidden Markov model)-profile based mapping available from the Pfam MySQL database. We then retained only the interactions between two ORFs whenever both ORFs contained Pfam domains that are seen to interact in iPfam. The resulting dataset comprises an intersection of iPfam and the APID-curated interactome. The annotation with Pfam domains entails a substantial filtering (from 14,437 APID-based interactions to 6,971 interactions) and hence represents a high-confidence network.

Orthologous classification and grouping of the annotated yeast ORFs (http://www.yeastgenome.org/) were determined from the clusters of ortholog groups [27]. Network representations were performed using standard routines from the program PAJEK [28].

### Quantifying backbone exposure of a protein chain.

Backbone exposure for node n, denoted Y_n_, is given as a percentage of contour length of the protein corresponding to under-protected residues, as defined below. The data were obtained from 488 yeast proteins (out of 6,199) reported in PDB complexes and four natively disordered yeast proteins [21]. The extent of backbone exposure at a particular residue was determined by counting the number of nonpolar carbonaceous side-chain groups contained within a 6.2 Å radius sphere (∼thickness of three water layers) centered at the α-carbon [17]. The extent of backbone shielding, η, within a structured region averaged over a nonredundant curated PDB database (1,662 proteins, free from redundancy and homology) is η = 14.2, with Gaussian dispersion = 7.2. Thus, a residue or backbone site with η < 7 is regarded as exposed. The statistics vary as other desolvation radii in the range 6Å < r < 7Å are adopted, but the tails of the distribution identify the same exposed residues. The structural integrity of soluble proteins requires that most backbone amides and carbonyls be protected from hydration. Thus, residues with absent backbone coordinates in a PDB entry (natively disordered [21,29]) are regarded as exposed and so are residues from entirely disordered proteins.

### Sequence-based inference of backbone exposure.

We adopt an established relationship between backbone exposure, η, and a structural parameter, λ_D_, that can be reliably determined from sequence: the propensity for inherent structural disorder in a region of a protein domain [17,29]. The latter parameter is assessed with a high degree of accuracy by the program PONDR-VLXT, a neural-network predictor of native disorder [29]. Thus, a disorder score λ_D_ (0 ≤ λ_D_ ≤ 1) is assigned to each residue within a sliding window. This value represents the predicted propensity of the residue to be in a disordered region (λ_D_ = 1 indicates full certainty). Only 6% of >1,100 nonhomologous PDB proteins give false positive predictions of disorder [17,29]. The correlation between propensity for disorder and wrapping implies that it is possible to predict backbone exposure directly from sequence. The correlation was originally established between the PONDR-VLXT score at a particular residue site and the extent of intramolecular protection, ρ, of the backbone hydrogen bond engaging that residue (if any). The latter quantity is operationally defined as ρ = η + η′, where η and η′ correspond to the two residues paired by the hydrogen bond. The strong correlation implies that we can infer the existence of residues with backbone exposure from the PONDR-VLXT score with 94% accuracy for regions with λ_D_ > 0.35. The correlation implies that the propensity to adopt a natively disordered state becomes pronounced for proteins that, because of their chain composition, cannot fulfill a minimal protection of their backbone hydrogen bonds.

## Supporting Information

Text S1 PDB-Reported Intra-Modular Hubs in Yeast Class \(11111) Supplementary results.
(121 KB PDF)

### Accession Numbers

The SwissProt (http://www.pir.uniprot.org/) numbers for the following yeast proteins/domains are in parentheses: SH3 Domain (P32790), Cytochrome c (Q753F4), Actin (P60010), Myosin V (Q04439), Ubiquitin (P61864), Calmodulin (P06787) and Rad14(P28519).

## Abbreviations

ORF: open reading frame
