# Supplementary material for: Molecular Basis for Evolving Modularity in the Yeast Protein Interaction Network
Source: PLoS Comput Biol. 2007 Nov 9;3(11):e226. doi: 10.1371/journal.pcbi.0030226 (PMC2065896; doi:10.1371/journal.pcbi.0030226)
Supplement: Supplementary file 1 [file pcbi.0030226.sd001.pdf]

Protocol S1
Supporting Data

Molecular basis for evolving modularity in the yeast protein interaction network

Ariel Fernández

Department of Bioengineering, Rice University,
Houston, TX 77005

Department of Computer Science, The University of Chicago,
Chicago, IL 60637

Phone: 713 348 3681; Fax: 713 348 3699; E-mail: arifer@rice.edu

I. PDB-reported intra-modular hubs in yeast class \(11111) *

(*) The class \(11111) is the complement of the ancestral class (11111) of proteins with orthologs in all life domains considered, and thus contains all the nodes incorporated to the ancestral network up to the present day.

SWISSPROT				PDB		
ID	ORF	Gene name	conn.	Code	Method	Chain
P02309	YNL030W	H4_YEAST	38	2FSB	Model	B/F=1-103
P02309	YNL030W	H4_YEAST	38	1ID3	X-ray	B/F=1-103
Q02821	YNL189W	IMA1_YEAST	30	2C1T	X-ray	A/B=88-541
Q02821	YNL189W	IMA1_YEAST	30	1WA5	X-ray	B=1-530
Q02821	YNL189W	IMA1_YEAST	30	1UN0	X-ray	A/B=88-530
Q02821	YNL189W	IMA1_YEAST	30	1EE5	X-ray	A=87-510
Q02821	YNL189W	IMA1_YEAST	30	1EE4	X-ray	A/B=87-509
Q02821	YNL189W	IMA1_YEAST	30	1BK6	X-ray	A/B=89-510
Q02821	YNL189W	IMA1_YEAST	30	1BK5	X-ray	A/B=89-510
P02829	YPL240C	HSP82_YEAST	30	2CGE	X-ray	A/B/D
P02829	YPL240C	HSP82_YEAST	30	2CG9	X-ray	A/B
P02829	YPL240C	HSP82_YEAST	30	2BRE	X-ray	A/B=1-219
P02829	YPL240C	HSP82_YEAST	30	2BRC	X-ray	A=1-214
P02829	YPL240C	HSP82_YEAST	30	2AKP	X-ray	A/B=25-210
P02829	YPL240C	HSP82_YEAST	30	1ZWH	X-ray	A=1-220
P02829	YPL240C	HSP82_YEAST	30	1USV	X-ray	A/C/E/G=272-530
P02829	YPL240C	HSP82_YEAST	30	1USU	X-ray	A=273-530
P02829	YPL240C	HSP82_YEAST	30	1US7	X-ray	A=1-214
P02829	YPL240C	HSP82_YEAST	30	1HK7	X-ray	A/B=273-560

P02829	YPL240C	HSP82_YEAST	30	1BGQ	X-ray	A
P02829	YPL240C	HSP82_YEAST	30	1AMW	X-ray	A=1-214
P02829	YPL240C	HSP82_YEAST	30	1AM1	X-ray	2-214
P02829	YPL240C	HSP82_YEAST	30	1AH8	X-ray	A/B=1-220
P02829	YPL240C	HSP82_YEAST	30	1AH6	X-ray	1-220
P02829	YPL240C	HSP82_YEAST	30	1A4H	X-ray	1-220
P13393	YER148W	TBP_YEAST	26	1YTF	X-ray	A=61-240
P13393	YER148W	TBP_YEAST	26	1YTB	X-ray	A/B=61-240
P13393	YER148W	TBP_YEAST	26	1TBP	X-ray	A/B=62-240
P13393	YER148W	TBP_YEAST	26	1TBA	NMR	B=61-240
P13393	YER148W	TBP_YEAST	26	1RM1	X-ray	A=1-240
P13393	YER148W	TBP_YEAST	26	1NH2	X-ray	A=61-240
P13393	YER148W	TBP_YEAST	26	1NGM	X-ray	A/E/I/M=61-240
Q06142	YLR347C	IMB1_YEAST	22	2BPT	X-ray	A=1-861
Q06142	YLR347C	IMB1_YEAST	22	2BKU	X-ray	B/D=1-861
P52286	YDR328C	SKP1_YEAST	22	1NEX	X-ray	A/C=1-194
P04050	YDL140C	RPB1_YEAST	21	2B8K	X-ray	A=1-1733
P04050	YDL140C	RPB1_YEAST	21	2B63	X-ray	A=1-1733
P04050	YDL140C	RPB1_YEAST	21	1Y77	X-ray	A=1-1733
P04050	YDL140C	RPB1_YEAST	21	1Y1Y	X-ray	A
P04050	YDL140C	RPB1_YEAST	21	1Y1W	X-ray	A=1-1733
P04050	YDL140C	RPB1_YEAST	21	1Y1V	X-ray	A=1-1733
P04050	YDL140C	RPB1_YEAST	21	1WCM		X-ray A=1-1733
P04050	YDL140C	RPB1_YEAST	21	1TWH	X-ray	A=1-1733
P04050	YDL140C	RPB1_YEAST	21	1TWG	X-ray	A=1-1733
P04050	YDL140C	RPB1_YEAST	21	1TWF	X-ray	A=1-1733
P04050	YDL140C	RPB1_YEAST	21	1TWC	X-ray	A=1-1733
P04050	YDL140C	RPB1_YEAST	21	1TWA	X-ray	A=1-1733
P04050	YDL140C	RPB1_YEAST	21	1SFO	X-ray	A=1-1733
P04050	YDL140C	RPB1_YEAST	21	1R9T	X-ray	A=1-1733
P04050	YDL140C	RPB1_YEAST	21	1R9S	X-ray	A=1-1733
P04050	YDL140C	RPB1_YEAST	21	1R5U	X-ray	A=1-1733
P04050	YDL140C	RPB1_YEAST	21	1PQV	X-ray	A=1-1733
P04050	YDL140C	RPB1_YEAST	21	1NT9	X-ray	A=1-1733
P04050	YDL140C	RPB1_YEAST	21	1NIK	X-ray	A=1-1733
P04050	YDL140C	RPB1_YEAST	21	1K83	X-ray	A=1-1733
P04050	YDL140C	RPB1_YEAST	21	1I6H	X-ray	A=1-1733
P04050	YDL140C	RPB1_YEAST	21	1I50	X-ray	A=1-1733
P04050	YDL140C	RPB1_YEAST	21	1I3Q	X-ray	A=1-1733
P06787	YBR109C	CALM_YEAST	16	1LKJ	NMR	A=2-147
P06787	YBR109C	CALM_YEAST	16	1F55	NMR	A=2-78
P06787	YBR109C	CALM_YEAST	16	1F54	NMR	A=2-78
P39517	YDL160C	DHH1_YEAST	15	1S2M	X-ray	A=31-425
P29468	YKR002W	PAP_YEAST	15	1FA0	X-ray	A/B=1-537
P39081	YDR228C	PCF11_YEAST	14	2BF0	X-ray	X=1-138
P39081	YDR228C	PCF11_YEAST	14	1SZA	X-ray	A/B/C=1-140
P39081	YDR228C	PCF11_YEAST	14	1SZ9	X-ray	A/B/C=1-140
P32628	YEL037C	RAD23_YEAST	14	1X3Z	X-ray	B=238-309
P32628	YEL037C	RAD23_YEAST	14	1X3W	X-ray	B=238-309
P25293	YKR048C	NAP1_YEAST	14	2AYU	X-ray	A=1-417

P17255	YDL185W	VATA_YEAST	14	1VDE	X-ray	A/B=284-737
P17255	YDL185W	VATA_YEAST	14	1UM2	X-ray	A/B=285-737, C/D=274-288
P17255	YDL185W	VATA_YEAST	14	1LWT	X-ray	A=284-737
P17255	YDL185W	VATA_YEAST	14	1LWS	X-ray	A=284-737
P17255	YDL185W	VATA_YEAST	14	1JVA	X-ray	A/B=274-747
P17255	YDL185W	VATA_YEAST	14	1GPP	X-ray	A=284-483
P17255	YDL185W	VATA_YEAST	14	1EF0	X-ray	A/B
P17255	YDL185W	VATA_YEAST	14	1DFA	X-ray	A=284-737
P06169	YLR044C	PDC1_YEAST	13	1QPB	X-ray	A/B=1-563
P06169	YLR044C	PDC1_YEAST	13	1PYD	X-ray	A/B=1-556
P06169	YLR044C	PDC1_YEAST	13	1PVD	X-ray	A/B=1-556
Q03330	YGR252W	GCN5_YEAST	12	1YGH	X-ray	A/B=99-262
Q03330	YGR252W	GCN5_YEAST	12	1E6I	X-ray	A=324-439
P41832	YNL271C	BNI1_YEAST	12	1Y64	X-ray	B=1327-1769
P41832	YNL271C	BNI1_YEAST	12	1UX5	X-ray	A=1350-1760
P41832	YNL271C	BNI1_YEAST	12	1UX4	X-ray	A/B=1352-1765
P38262	YBR103W	SIF2_YEAST	12	1R5M	X-ray	A=113-535
P22336	YAR007C	RFA1_YEAST	12	1YNX	NMR	A=181-294
P14907	YJL041W	NSP1_YEAST	12	1O6O	X-ray	D/E/F=497-608
P11433	YAL041W	CDC24_YEAST	12	1Q1O	NMR	A=761-854
P11433	YAL041W	CDC24_YEAST	12	1PQS	NMR	A=778-854
P06782	YDR477W	SNF1_YEAST	12	2FH9	X-ray	A=46-319
P06782	YDR477W	SNF1_YEAST	12	2EUE	X-ray	A/B=41-315
P04147	YER165W	PABP_YEAST	12	1IFW	NMR	A=489-577
P29366	YBR200W	BEM1_YEAST	11	1IPG	NMR	A=472-551
P29366	YBR200W	BEM1_YEAST	11	1IP9	NMR	A=472-551
P10081	YJL138C	IF4A_YEAST	11	1QVA	X-ray	A=1-224
P10081	YJL138C	IF4A_YEAST	11	1QDE	X-ray	A=9-232
P10081	YJL138C	IF4A_YEAST	11	1FUU	X-ray	A/B=1-395
P10081	YJL138C	IF4A_YEAST	11	1FUK	X-ray	A=231-395
P34162	YHR041C	MED20_YEAST	10	2HZS	X-ray	A/C/E/G=2-210
P34162	YHR041C	MED20_YEAST	10	2HZM	X-ray	A/C/E/G=1-210
P16892	YBL016W	FUS3_YEAST	10	2FA2	X-ray	A/B=1-353
P16892	YBL016W	FUS3_YEAST	10	2F9G	X-ray	A=1-353
P16892	YBL016W	FUS3_YEAST	10	2F49	X-ray	A=1-353
P16892	YBL016W	FUS3_YEAST	10	2B9J	X-ray	A=1-353
P16892	YBL016W	FUS3_YEAST	10	2B9I	X-ray	A=1-353
P16892	YBL016W	FUS3_YEAST	10	2B9H	X-ray	A=1-353
P16892	YBL016W	FUS3_YEAST	10	2B9F	X-ray	A=1-353
P54784	YML065W	ORC1_YEAST	9	1ZHI	X-ray	A=1-219
P54784	YML065W	ORC1_YEAST	9	1ZBX	X-ray	A=1-219
P54784	YML065W	ORC1_YEAST	9	1M4Z	X-ray	A/B=1-235
P22216	YPL153C	RAD53_YEAST	9	2A0T	NMR	A=14-164
P22216	YPL153C	RAD53_YEAST	9	1QU5	NMR	A=549-730
P22216	YPL153C	RAD53_YEAST	9	1K3Q	NMR	A=14-164
P22216	YPL153C	RAD53_YEAST	9	1K3N	NMR	A=14-164
P22216	YPL153C	RAD53_YEAST	9	1K3J	NMR	A=14-164
P22216	YPL153C	RAD53_YEAST	9	1K2N	NMR	A=573-730
P22216	YPL153C	RAD53_YEAST	9	1K2M	NMR	A=573-730

P22216	YPL153C	RAD53_YEAST	9	1J4Q	NMR	A=14-164
P22216	YPL153C	RAD53_YEAST	9	1J4P	NMR	A=14-164
P22216	YPL153C	RAD53_YEAST	9	1J4O	NMR	A=14-164
P22216	YPL153C	RAD53_YEAST	9	1J4L	NMR	A=573-730
P22216	YPL153C	RAD53_YEAST	9	1J4K	NMR	A=573-730
P22216	YPL153C	RAD53_YEAST	9	1G6G	X-ray	A/B=29-155
P22216	YPL153C	RAD53_YEAST	9	1G3G	NMR	A=2-164
P22216	YPL153C	RAD53_YEAST	9	1FHR	NMR	A=573-730
P22216	YPL153C	RAD53_YEAST	9	1FHQ	NMR	A=573-730
P22216	YPL153C	RAD53_YEAST	9	1DMZ	NMR	A=573-730
P17555	YNL138W	CAP_YEAST	9	1KQ5	X-ray	A/B=369-526
P17555	YNL138W	CAP_YEAST	9	1K4Z	X-ray	A/B=369-526
Q08649	YOR244W	ESA1_YEAST	8	1MJB	X-ray	A=160-435
Q08649	YOR244W	ESA1_YEAST	8	1MJA	X-ray	A=160-435
Q08649	YOR244W	ESA1_YEAST	8	1MJ9	X-ray	A=160-435
Q08649	YOR244W	ESA1_YEAST	8	1FY7	X-ray	A=160-435
Q08278	YOL135C	MED7_YEAST	8	1YKH	X-ray	A=102-205
Q08278	YOL135C	MED7_YEAST	8	1YKE	X-ray	A/C=102-205
P38304	YBR193C	MED8_YEAST	8	2HZS	X-ray	I/J/K/L=190-216
P34087	YDR404C	RPB7_YEAST	8	2B8K	X-ray	G=1-171
P34087	YDR404C	RPB7_YEAST	8	2B63	X-ray	G=1-171
P34087	YDR404C	RPB7_YEAST	8	1Y77	X-ray	G=1-171
P34087	YDR404C	RPB7_YEAST	8	1Y1Y	X-ray	G
P34087	YDR404C	RPB7_YEAST	8	1Y1W	X-ray	G=1-171
P34087	YDR404C	RPB7_YEAST	8	1Y1V	X-ray	G=1-171
P34087	YDR404C	RPB7_YEAST	8	1Y14	X-ray	B/D=1-171
P34087	YDR404C	RPB7_YEAST	8	1WCM		X-ray G=1-171
P34087	YDR404C	RPB7_YEAST	8	1PQV	X-ray	G=1-171
P34087	YDR404C	RPB7_YEAST	8	1NT9	X-ray	G=1-171
P32776	YDR311W	TFB1_YEAST	8	2GS0	NMR	A=1-115
P32776	YDR311W	TFB1_YEAST	8	1Y5O	NMR	A=2-115
P25454	YER095W	RAD51_YEAST	8	1SZP	X-ray	A/B/C/D/E/F=81-400
P23561	YLR362W	STE11_YEAST	8	1X9X	NMR	A/B=37-104
P23561	YLR362W	STE11_YEAST	8	1OW5	NMR	A=36-113
P18851	YOR212W	GBB_YEAST	8	1SCG	Model	B=1-423
P16649	YCR084C	TUP1_YEAST	8	1ERJ	X-ray	A/B/C=282-713

II. Variation in functional composition with network evolution

To fully characterize the evolutionary trend towards increasing modularity, the functional composition of the ancient network is contrasted against the present-day network, using the genetic ontology (GO) Slim classification of open reading frames [1]. The functional distribution differences associated with evolutionary change are shown in Fig. S1. We find that the relative representation of certain functional categories persists throughout evolutionary change, in spite of the drastic reduction in number of proteins. Furthermore, dominant

functionalities that become significant in the ancient network may be identified by a representativity larger than twice the basal value 3.5% of the corresponding representativity of the functional class in the present-day proteome. The basal value corresponds to the fact that the ancient proteome contains 3.5% of present-day proteins. Thus, dominant functional classes in the ancient network are: ribosome, RNA metabolism, transcription, nuclear organization, signal transduction and stress response.

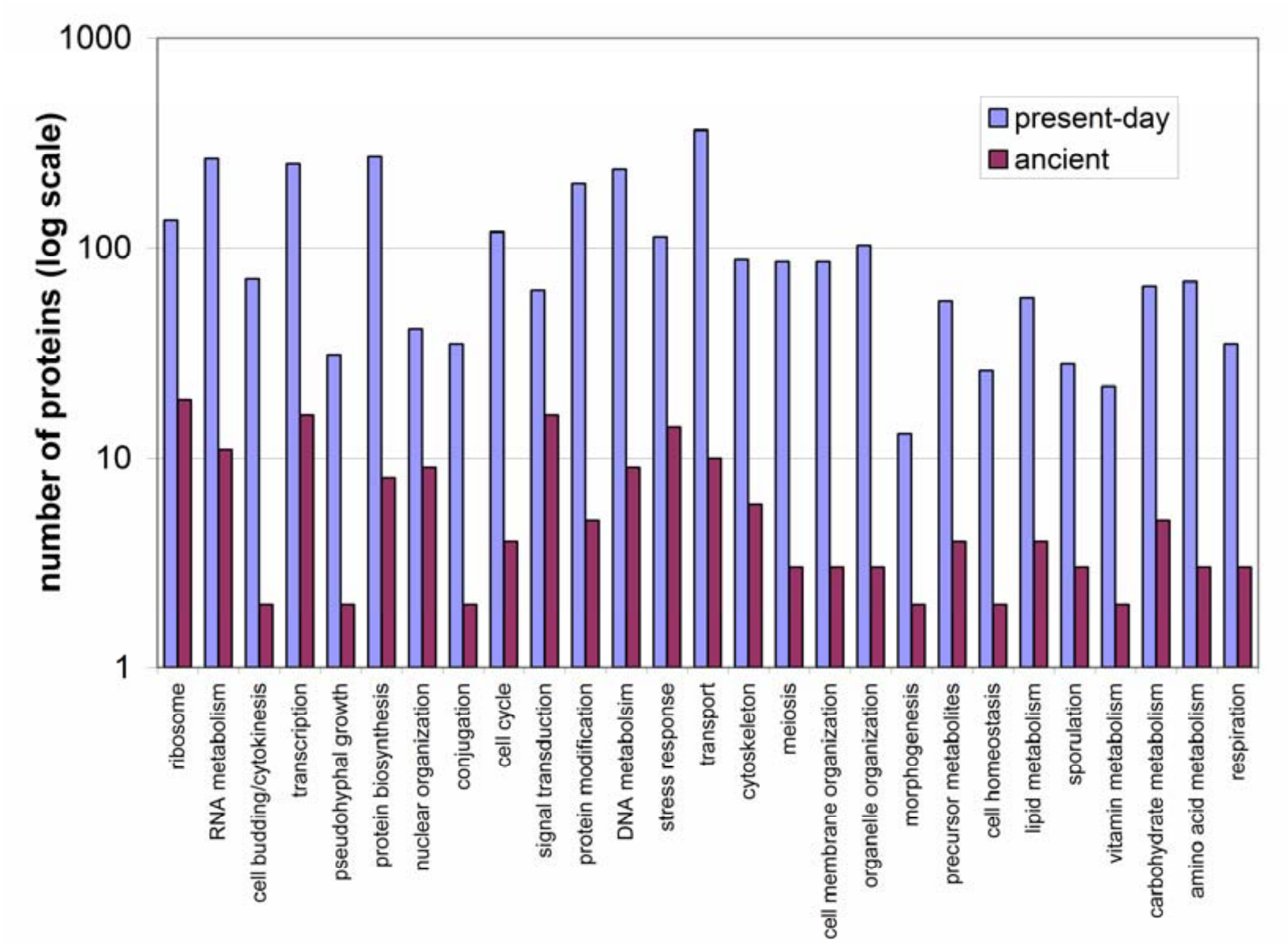

Figure S1. Functional composition of present-day and ancient proteome. The classification used the slim gene ontology (GO).

III. Supplementary results on the ortholog-based trimming procedure

Our analysis of the topological evolution of modularity hinges on the validity of a trimming procedure based on the retention of nodes according to their extent of orthologous representation. This procedure is supported by recent analysis [2], yet it is somewhat at odds with the estimation by Wagner [3] that interactions in yeast are lost at a rate of 2.2×10^{-3}/million year. This assessment uses the extent of synonymous substitution as surrogate for evolutionary time and adopts high-throughput two-hybrid experiments to map protein interactions. The latter data is known to be unreliable and plagued by false positives [4] and would require a careful curation which was not perform at the time of the analysis. Furthermore, to assess the

loss of interactions, one would need to perform a structural analysis to verify whether indeed the protein interfaces have been modified or have coevolved through evolutionary change, which is not likely to be uniform across the protein chain. This analysis was not carried out in [3]. Most importantly, the assertion on the loss of interactions is at odds with the established high level of conservation of functionally competent protein interfaces [5, 6].

1. Prachumwat A, Li W-H (2006) Protein function, connectivity and duplicability in yeast. Mol Biol Evol 23: 30-39
2. Qin H, Lu H S, Wu W B, Li W-H (2003) Evolution of the yeast protein interaction network. Proc Natl Acad Sci USA 100: 12820-12824
3. Wagner A (2001) The yeast protein interaction network evolves rapidly and contains few redundant duplicate genes. Mol Biol Evol 18: 1283-1292
4. Krogan D, Cagney G, Yu H, Zhong G, Guo X, *et al*. (2006) Global landscape of protein complexes in the yeast *Saccharomyces cerevisiae*. Nature 440: 637-643
5. Fernández A, Berry R S (2004) Molecular dimension explored in evolution to promote proteomic complexity. Proc Natl Acad Sci USA 101: 13460-13465
6. Kim P M, Lu L J, Xia Y, Guerstein M B (2006) Relating three-dimensional structures to protein networks provides evolutionary insights. Science 314: 1938-1941
